# The effect of mutation subtypes on the allele frequency spectrum and population genetics inference

**DOI:** 10.1093/g3journal/jkad035

**Published:** 2023-02-10

**Authors:** Kevin Liao, Jedidiah Carlson, Sebastian Zöllner

**Affiliations:** Department of Biostatistics, University of Michigan, Ann Arbor, MI 48109, USA; Department of Integrative Biology, University of Texas at Austin, Austin, TX 78712, USA; Department of Population Health, University of Texas at Austin, Austin, TX 78712, USA; Department of Biostatistics, University of Michigan, Ann Arbor, MI 48109, USA; Department of Psychiatry, University of Michigan, Ann Arbor, MI 48109, USA

**Keywords:** allele frequency spectrum, mutation types, population genetics

## Abstract

Population genetics has adapted as technological advances in next-generation sequencing have resulted in an exponential increase of genetic data. A common approach to efficiently analyze genetic variation present in large sequencing data is through the allele frequency spectrum, defined as the distribution of allele frequencies in a sample. While the frequency spectrum serves to summarize patterns of genetic variation, it implicitly assumes mutation types (A→C vs C→T) as interchangeable. However, mutations of different types arise and spread due to spatial and temporal variation in forces such as mutation rate and biased gene conversion that result in heterogeneity in the distribution of allele frequencies across sites. In this work, we explore the impact of this simplification on multiple aspects of population genetic modeling. As a site’s mutation rate is strongly affected by flanking nucleotides, we defined a mutation subtype by the base pair change and adjacent nucleotides (e.g. AAA→ATA) and systematically assessed the heterogeneity in the frequency spectrum across 96 distinct 3-mer mutation subtypes using *n* = 3556 whole-genome sequenced individuals of European ancestry. We observed substantial variation across the subtype-specific frequency spectra, with some of the variation being influenced by molecular factors previously identified for single base mutation types. Estimates of model parameters from demographic inference performed for each mutation subtype’s AFS individually varied drastically across the 96 subtypes. In local patterns of variation, a combination of regional subtype composition and local genomic factors shaped the regional frequency spectrum across genomic regions. Our results illustrate how treating variants in large sequencing samples as interchangeable may confound population genetic frameworks and encourages us to consider the unique evolutionary mechanisms of analyzed polymorphisms.

## Introduction

The advent of whole-genome sequencing in the past decade has transformed the field of population genetics and allowed for a host of new analyses on genetic variation both within and between populations (Wong *et al.* 2013; Gudbjartsson *et al.* 2015; The 1000 Genomes Project Consortium *et al.* 2015; Mallick *et al.* 2016; Taliun *et al.* 2021). As a result, this abundance of information has allowed for a host of methods to infer population genetic parameters such as mutation rates, demographic history, natural selection, and more (Pool *et al.* 2010; Li and Durbin 2011; Schiffels and Durbin 2014; Carlson *et al.* 2018; Aikens *et al.* 2019). One class of methods, that in recent years have regained popularity for population genetics inference due to their computational tractability, are based on the allele frequency spectrum (AFS) (Nielsen 2000; Williamson *et al.* 2005; Gutenkunst *et al.* 2009; Ronen *et al.* 2013; Kamm *et al.* 2020). The AFS is defined as the distribution of allele frequencies at segregating sites in a sample and serves as a summary statistic of the genetic variation within that population (Fisher 1931; Ewens 1972). As the AFS ignores information on linkage between sites by simply capturing the frequency of derived alleles in a sample, it effectively reduces genome-wide data for large samples into a single distribution. As a result, population genetics methods based on the AFS allow for analyzing millions of variable sites in thousands of individuals (Gutenkunst *et al.* 2009; Ronen *et al.* 2013).

Current AFS-based methods to test for selection (using the local AFS in a genomic region) and infer demographic history (using the genome-wide AFS) use a frequency spectrum constructed from all segregating sites in a sample. These AFS-based methods can generally be grouped into two categories: (1) methods which reduce the high-dimensional AFS to a one-dimensional summary statistic such as Tajima’s D, Fu and Li’s D and F, and Fay and Wu’s H (Tajima 1989; Fu and Li 1993; Fay and Wu 2000; Marth *et al.* 2004) and (2) methods that model the full AFS such as *δ*a*δ*i, momi, and SFselect (Gutenkunst *et al.* 2009; Ronen *et al.* 2013; Kamm *et al.* 2017). Each of these methods leverage that selection and demographic history affect the shape of the frequency spectrum. Thus, comparing the observed AFS to the expected AFS under a neutral population or a particular demographic model can be used to test for selection or estimate demographic model parameters.

Typical construction of the AFS to summarize data and conduct inference treats all sites equally. However, the AFS can differ between sites due to heterogeneity in evolutionary forces. Across sites, mutation rates vary driven primarily by immediate surrounding sequence context and local genomic factors (Zhang *et al.* 2007; Carlson *et al.* 2018; Aikens *et al.* 2019) (e.g. CpG TpG sites have orders of magnitude higher rates due to methylation). For fast mutating sites, recurrent mutations (i.e. multiple independent mutation events) violate the infinite sites model (assumes each site is equally likely to mutate and will only mutate once) and lead to multiple carriers of the same allele. Thus, in large samples, fast mutating sites (Jenkins and Song 2011; Harpak *et al.* 2016; Seplyarskiy *et al.* 2021; Wakeley *et al.* 2022) have a general shift away from rarer frequencies as two or more lower count mutations occurring at the same position are evaluated as a single higher count mutation (e.g. two singletons treated as one doubleton). While it is possible for the opposite scenario where an additional mutation reverses the original, such backwards mutations occur at a much lower rate. Moreover, empirical findings (Harpak *et al.* 2016) suggest the scenario of shifting to higher allele counts is more prevalent in shaping the AFS. Another factor non-uniformly shaping the AFS across sites is biased gene conversion (gBGC), which occurs during recombination and is the process in which A/T – G/C heterozygotes have preferential transmission of the G/C allele. In highly recombining regions, gBGC leads to increases in the allele frequencies of A/T → G/C mutation types (Duret and Galtier 2009; Kostka *et al.* 2012; Lachance and Tishkoff 2014) and is known to mimic the effect of positive selection on the AFS (right shifting the AFS). Due to these evolutionary forces uniquely shaping the AFS across different sites, combining all sites into a single overall AFS (as is typically done) may bias inference as signals of selection or demographic history in the overall AFS are confounded by its composition of sites. This confounding will likely increase as genetic sample sizes grow larger and any AFS heterogeneity across sites is exacerbated. However, the impact of this potential confounding on population genetics inference is currently unknown.

In this work, we combined and extended these known drivers to study how the AFS varies across mutation subtypes and the downstream implications on population genetics inference. We used a collection of 53,133,922 SNPs from 3,556 sequenced individuals from the Bipolar Research in Deep Genome and Epigenome Sequencing (BRIDGES) study (Carlson *et al.* 2018). Each variant was classified into one of 96 mutation subtype 3-mers defined by the specific point mutation and its immediate adjacent bases. For each subtype, we constructed an AFS to effectively partitioning the overall AFS into 96 distinct frequency spectra. Under the infinite sites model, these 96 AFSs should differ only due to sampling variation. We showed that the AFS differs widely between subtypes, even outside CpG TpG sites, with much of these differences being driven by mutation rate heterogeneity and biased gene conversion, two factors previously implicated to shape the AFS at the single base level. To disentangle the two factors, we further derived a novel Tajima’s D-type statistic *D*_−2_ that removes the singleton and doubleton contribution. As a result of AFS heterogeneity across subtypes, theoretical inference of demographic history using the full-genome wide AFS for a single subtype under a growth and three-epoch model varied drastically among the subtypes. Similarly, in local genomic regions we found both the local composition of subtypes and genomic factors, such as recombination rate, to be significant predictors of the regional AFS across the genome.

## Materials and methods

We analyzed whole-genome sequencing data from the Bipolar Research in Deep Genome and Epigenome Sequencing (BRIDGES) study of unrelated individuals of European ancestry. The samples were aggregated from a variety of studies that each collected cases and controls of individuals with Bipolar Disorder (Carlson *et al.* 2018). Sequencing was performed, per the Illumina protocol on Build GRCh37, to generate our final dataset with a mean coverage of 9.6x across individuals. After filtering out samples with high contamination, case misspecification, ancestry outliers, and relatedness our final sample included 3,556 unrelated European individuals with a total of 56,482,865 variants.

Each of the single-nucleotide polymorphisms (SNPs) observed in our dataset was classified as one of six mutation types, determined by the ancestral allele and the derived allele: A→C, A→G, A→T, C→A, C→G, C→T. We determined the ancestral allele using the 1000 Genomes ancestral alleles for Build 37 and annotated via bcftools (Danecek *et al.* 2011; The 1000 Genomes Project Consortium *et al.* 2015). Note that each mutation type is defined to account for the complementary nature of DNA, and thus we ignored which strand the mutation occurs on (e.g. an SNP with ancestral allele T and derived allele G corresponds to an A→C SNP on the opposite strand and vice versa, so for brevity, we classify both as A→C mutations). Each mutation type was further refined into 3-mer mutation subtypes by considering the surrounding immediate nucleotides using the GRCh37 human reference sequence. This resulted in 96 distinct mutation subtypes for our analysis (4 possible bases downstream * 6 mutation types * 4 possible bases upstream = 96 mutation subtypes). In previous work (Carlson *et al.* 2018), we estimated relative mutation rates for each 3-mer using singletons from the same BRIDGES dataset.

### Comparison of the AFS across mutation subtypes to identify signals of evolutionary forces driving AFS heterogeneity

We first constructed a distinct unfolded AFS for each of the 96 mutation subtypes. For a given haploid sample of size *n*, let *η*_*i*_ be the number of segregating sites in the sample in which exactly *i* individuals have the derived allele. The AFS is then defined by the vector (*η*_1_, *η*_1_, …, *η*_*n*−1_).

We summarized and compared the 96 subtype AFSs using the ratio of singletons to doubletons and Tajima’s D (Tajima 1989). The ratio of singletons to doubletons $\left(\frac{\eta_{1}}{\eta_{2}}\right)$ was used to identify signals of recurrent mutations lowering the singleton count (Harpak *et al.* 2016) for sites with higher mutation rates, where the ratio reflected any reduction in singletons and increase in doubletons. While recurrent mutations work to shift the frequency for many of the rarest frequency variants in high mutation rate sites, their effect is most prevalent in shifting two singletons to a single doubleton count (Jenkins and Song 2011; Harpak *et al.* 2016; Wakeley *et al.* 2022). Tajima’s D was used to identify signals of biased gene conversion, where Tajima’s D is a summary statistic of the high-dimensional AFS computed by comparing two unbiased estimates of *θ* = 4*Nμ* (*N* is effective diploid population size and *μ* is mutation rate) under a neutral population model: Watterson’s estimator *θ*_*W*_ and Mean Pairwise Difference $\theta_{\pi }$:


D=θπ−θWvar(θπ−θW)θπ=(n2)−1[∑i=1n−1ηii(n−i)]θW=Shn

Here*S* is the number of segregating sites, *n* is the haploid sample size, and $h_{n}=\sum_{i=1}^{n-1}\frac{1}{i}$. *θ*_*π*_ assigns more weight to alleles segregating at intermediate counts compared to *θ*_*W*_, which weights all allele counts equally (Korneliussen *et al.* 2013). As a result, an excess of rare or intermediate frequency alleles in the AFS, can be observed in a more negative or positive Tajima’s D. As gBGC skews allele frequencies towards intermediate frequency variants for weak to strong mutation types (A/T → C/G) and towards rare variants for strong to weak mutation types (Lachance and Tishkoff 2014), we expect weak to strong mutation types to have a “more positive” Tajima’s D and vice versa for strong to weak types.

As Tajima’s D is strongly influenced by the singleton and doubleton count, we derived a novel D statistic, which we call *D*_−2_, that removes the singleton *η*_1_ and doubleton *η*_2_ contribution to Tajima’s D:


D=θπ−2−θW−2var(θπ−2−θW−2)θπ−2=n(n−1)(n−2)(n−3)(n2)−1{[∑i=1n−1ηii(n−i)]−η1(n−1)−2η2(n−2)}θW−2=S−η1−η2hn−32

Inthe numerator, *θ*_*π*−2_ and *θ*_*W*−2_ are the Mean Pairwise Difference and Watterson’s estimators with *η*_1_ and *η*_2_ removed and then reweighted to ensure both estimators stay unbiased for *θ* under a neutral population. Thus, the *D*_−2_ statistic allowed us to summarize the AFS for all other allele counts in a familiar form to investigate the effects of gene conversion on the AFS without potential confounding of the singleton/doubleton count driven by recurrent mutations in sites with higher mutation rates. We derived the analytical form for the variance of the difference in *θ*_*π*−2_ and *θ*_*W*−2_ using covariance derivations for linear combinations of the AFS (Fu 1995; Durrett 2008) (Supplementary File S2). To check the behavior of our statistic under the null, we simulated neutral frequency spectra for two separate subtype’s estimate of *θ* using fastsimcoal2 (Excofffier *et al.* 2021) (Supplementary File S2). Similar to Tajima’s D, comparisons of our *D*_−2_ statistic across subtypes were used to interrogate potential signals of gBGC.

### Effect of heterogeneity in the genome-wide AFS across subtypes on demographic inference

**Fig. 1.**
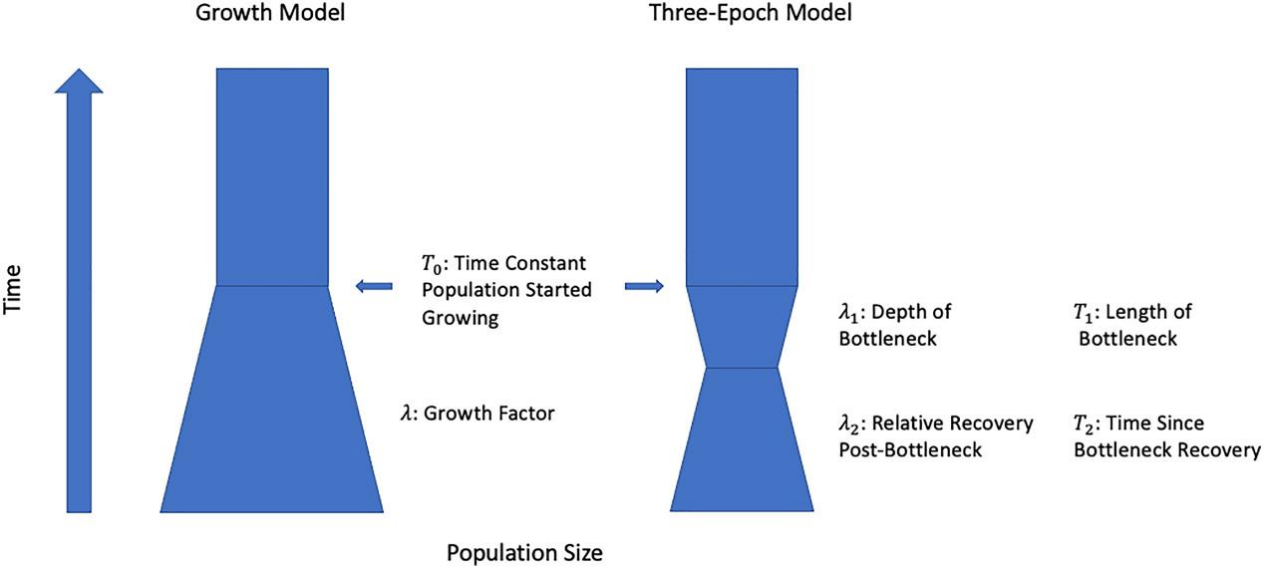
Diagram of growth and three-epoch demographic models fit in *δaδi*. Parameters of growth model include time since ancestral constant size population started growing *T*_0_ and growth factor *λ*. Parameters of three-epoch model include length of bottleneck *T*_1_, time since bottleneck recovery *T*_2_ as well as bottleneck depth *λ*_1_ and recovery *λ*_2_. Each model fit separately using the 96 distinct subtype AFS.

To assess the effect of AFS heterogeneity across mutation subtypes on demographic inference we used the method *δaδi* (Gutenkunst *et al.* 2009), which takes a diffusion approach to simulate the AFS for a predefined demographic model. Once simulated, comparisons to the observed genome-wide AFS allow *δ*a*δ*i to infer parameters for the given demographic model (Gutenkunst *et al.* 2009). Here, we inferred demographic model parameters separately for each of the 96 genome-wide AFS across mutation subtypes to assess systematic differences. For each *δaδi* run, we considered two models of population history. In the first model, we modeled a population undergoing exponential growth (Fig. 1), with a constant ancestral effective population size *N*_*e*_ that started exponentially growing at some time *T*_0_ in the past to a present population size *λN*_*e*_ while mutations accumulate with rate *μ*. In the second model, we considered a modified three-epoch model (Fig. 1), a more natural model for the human population that allows for two changes of population size. We model a population with an ancestral population size *N*_*e*_ that at time *T*_0_ contracts in a bottleneck of length *T*_1_ to size *λ*_1_*N*_*e*_ and recovers to relative size *λ*_2_*N*_*e*_ during time *T*_2_. Under both models, we inferred (1) the compound parameter *θ* = 4*N*_*e*_*μ*, (2) times *T*_0_ (both models) and *T*_1_, *T*_2_(three-epoch model) in generations, and (3) the ratios between the ancestral and post-change population sizes *λ* (growth), *λ*_1_, *λ*_2_ (three-epoch). We computed the ancestral effective population size solving $N_{e}=\frac{\theta }{4\mu }$ where the absolute mutation rate was derived from extremely rare variants in the same BRIDGES dataset (Carlson *et al.* 2018). To derive an absolute per-site, per-generation mutation rate from the relative mutation rate, we assume 60 de novo mutations per generation (a value typically observed in trio studies) (Kong *et al.* 2012; Francioli *et al.* 2015):


$$\mu_{subtype}=RelRate_{subtype}*\frac{60}{\sum_{subtypes}NumMotifs_{subtype}*RelRate_{subtype}}$$

Followingauthor’s guidelines, we ran *δ*a*δ*i for each subtype 10 separate times, perturbing starting parameters to determine the best-fit model (via log likelihood) and lower and upper bounds of optimization (widening if estimated parameters were near the boundaries). Under the growth model, we used bounds for *T*_0_ to be [0, 10] and *λ* to be [0, 750] with starting values of 3 and 150, respectively. Similarly, for the three-epoch model we used bounds for *λ*_1_ to be [0, 1] to enforce a bottleneck, *λ*_2_ to be [0, 150], *T*_1_ to be [0, 10], and *T*_2_ to be [0, 10]. For each model, we estimated standard errors for model parameters using 100 bootstrapped AFS of each mutation subtype. We used *δ*a*δ*i’s implementation of the Godambe Information Matrix, which approximates parameter uncertainties as normal to avoid the task of fitting each bootstrapped AFS.

### Effect of heterogeneity in the local composition of mutation subtypes on the regional AFS

**Fig. 2.**
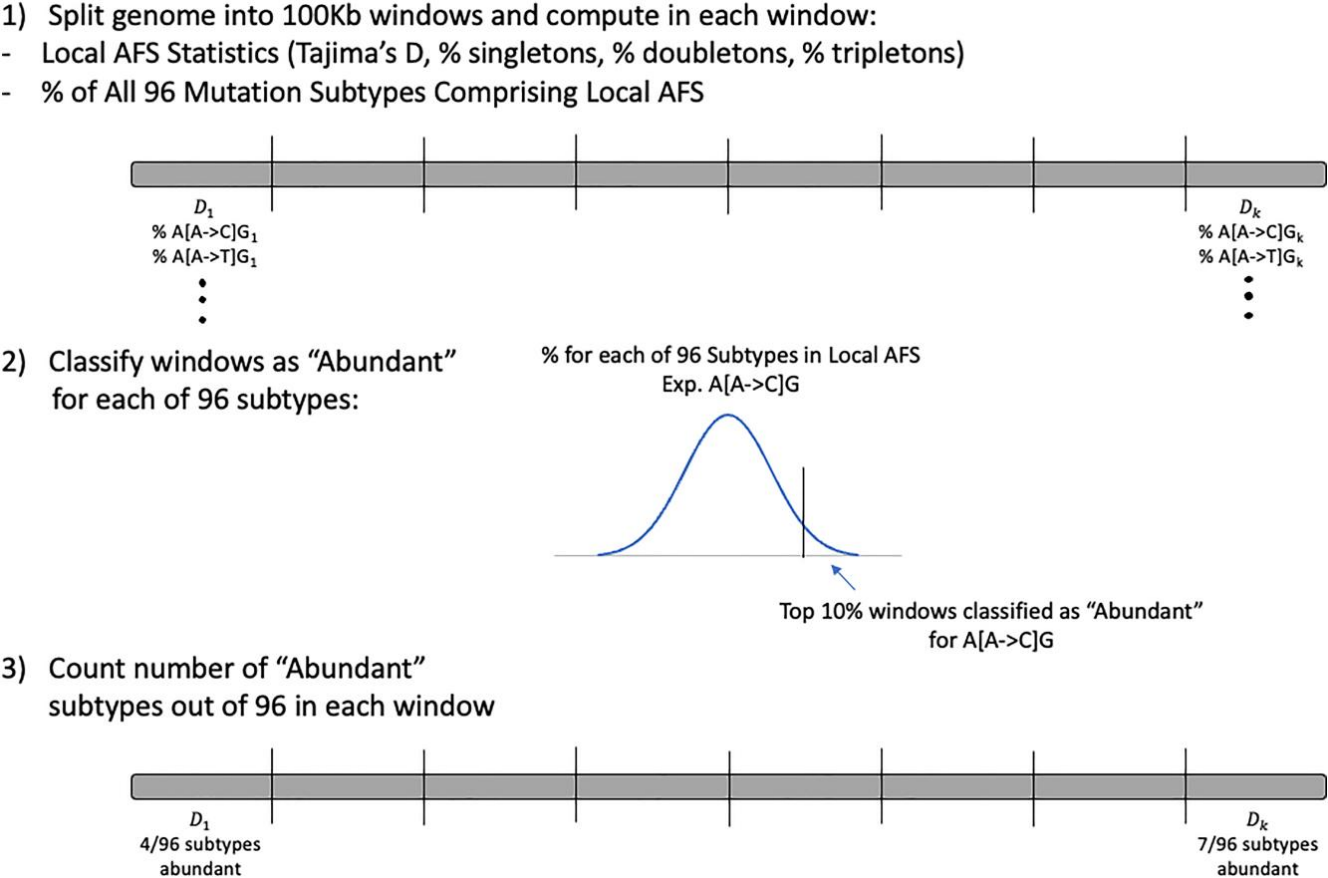
Diagram of analysis to assess local mutation subtype composition and regional AFS. In 100 kb windows, we compute (1) local AFS statistics (Tajima’s D, % singletons, etc.), (2) the counts and proportions comprising the local AFS for each of 96 mutation subtypes. Windows in the top 10% of subtype proportion across the genome are classified as “abundant” for that subtype and we count the number of abundant subtypes in each window.

Practical applications of AFS-based statistics (such as Tajima’s D) often use the combined local AFS (i.e. derived from all segregating sites, regardless of subtype) as it varies over non-overlapping windows across the genome. We evaluated how the local AFS could be shaped by heterogeneity in its composition of mutation subtypes. To reduce the potential confounding of selection when assessing the relationship between local region subtype composition and the AFS, we subset sites to only intergenic sites [assuming limited selection on intergenic regions (Ponting and Lunter 2006; Asthana *et al.* 2007)] annotated using EPACTs (Kang 2014). We partitioned the remaining genome in 100 kb windows and computed in each window from the local AFS (1) Tajima’s D, (2) the proportion of overall singletons, doubletons, and tripletons and (3) the counts/proportion of each of the 96 subtypes comprising the local AFS. For each subtype, we then ranked windows according to proportion constituting the local AFS and classified a window as being “abundant” in that subtype if its proportion fell in the top 10% of windows (Fig. 2).

We first evaluated whether the distribution of Tajima’s D across these windows was independent of the distribution of variants by stratifying windows into 10 quantiles by Tajima’s D and finding the mean number of abundant subtypes out of 96 in each quantile. Under the null expectation that the composition of mutation subtypes in a local AFS is independent of the Tajima’s D in a window, we would expect the number of abundant subtypes to be constant across quantiles. We further stratified abundant subtypes by whether these subtypes were (1) low vs high mutation rate using the median mutation rate across subtypes as the separator and (2) direction of gene conversion by WS, SW, and indifferent to interrogate whether mutation rate heterogeneity or biased gene conversion drove the dependence in the distribution of local Tajima’s D and variants.

We quantified the overall contribution of local subtype heterogeneity on the observed local AFS statistics (Tajima’s D, % singletons, etc.) by computing the expected value of each statistic. Using the count of each subtype as a weight, we computed the expected local AFS statistic in a window as a weighted mean of the 96 subtype’s genome wide observed value. For example, the expected Tajima’s D in a window is:


$$D_{expected}=\frac{\sum_{i=1}^{96}D_{GW}^{i}w_{i}}{\sum_{i=1}^{96}w_{i}}$$

Where$D_{GW}^{i}$ is Tajima’s D using the genome-wide AFS for subtype *i* and the weights *w*_*i*_ are the number of subtypes *i* in the window. If local heterogeneity in mutation subtypes fully explained regional differences in a statistic, we would expect observed and expected statistic values to be equal. To further quantify the contribution of local subtype heterogeneity, we directly regressed each of the observed local AFS statistic on the expected statistic (while adjusting for potential confounders) using a multivariate generalized estimating equation (GEE) linear model with a working exchangeable correlation structure for each chromosome. For example, modeling the local observed Tajima’s across the genome:


$$\begin{array}{rl} D_{obs} & =\beta_{0}+\beta_{exp}D_{exp}+\beta_{RR}RR+\beta_{GC}GC\\ R(\rho ) & =\left[\begin{array}{ccc} 1 & \ldots & \rho \\ \vdots & 1 & \vdots \\ \rho & \ldots & 1 \end{array}\right] \end{array}$$

Where*D*_*exp*_, *RR*, and *GC* are the expected local Tajima’s D, recombination rate and % GC content in the window, *β*_*exp*_,*β*_*RR*_,*β*_*GC*_ are the respective effects, and *R*(*ρ*) is the working exchangeable correlation structure. We adjust for recombination rate and GC content in a window because recombination rate is known to confound selection (O’Reilly *et al.* 2008; Glémin *et al.* 2015), and GC content affects germline mutation rates (Smith *et al.* 2002). The GEE framework was used to produce robust standard errors because neighboring genomic regions have correlated statistics (i.e. Tajima’s D) and thus would affect the standard error estimates of an ordinary linear regression.

## Results

We leveraged large sample whole-genome sequencing to first evaluate patterns in the overall AFS and assess potential heterogeneity in the AFS across mutation subtypes. In the overall genome-wide AFS, among the 56,482,865 total SNPs across *N* = 3, 556 samples, we observed an excess of rare variation shown through the proportion of singletons (60.3%), doubletons (9.91%), and tripletons (4.01%) (Table 1). This excess of rare variation is consistent with exponential and accelerating faster than exponential population growth in recent human demographic history (Coventry *et al.* 2010; Keinan and Clark 2012; Reppell *et al.* 2014). When partitioning the overall AFS by the six mutation types, the proportion of singletons varied greatly with C→A mutation types having the highest singleton proportion (63.7%) and C→T mutation types having the lowest (58.7%) (Table 1). We observed additional variation in the AFS when considering mutation types on a more granular scale through stratifying by flanking nucleotides (Tables 1 and 2). For example, we found the A[C→T]G mutation subtype, a CpG TpG site with an outlier mutation rate, had considerably lower singleton and higher doubleton proportions (53.82%, 13.68%) when compared to A[C→T]A (61.93%, 9.67%), A[C→T]C (59.29%, 9.53%), and A[C→T]T (60.86%, 9.51%). Notably, even outside the CpG TpG subtype, some singleton proportions of the same mutation type differ by >2% across subtypes when altering the +1 base (C vs A). Due to our large sample sizes, differences in the listed singleton and proportions across the four A[C→T]X subtypes were highly significant after adjusting for multiple comparisons (*P* < 0.001). Counts and proportions for the other 92 3-mer subtypes can be found in Supplementary File S1.

**Table 1.** (a) Genome-wide counts and proportions of singletons, doubletons, and tripletons for six single base mutation types. (b) Genome-wide counts and proportions of singletons, doubletons, and tripletons for A[C→T]X subtypes varying the base downstream.

Mutation type	# Sites	Singletons (%)	Doubletons (%)	Tripletons (%)
A→C	4,187,865	61.36	9.29	3.77
A→G	16,132,320	60.15	9.45	3.85
A→T	4,020,474	61.50	9.35	3.86
C→A	5,944,362	63.69	9.11	3.68
C→G	4,898,261	62.26	9.43	3.82
C→T	22,678,843	58.67	10.70	4.31
Overall	53,133,922	60.30	9.91	4.01
A[C→T]A	1,636,849	61.93	9.67	3.91
A[C→T]C	1,026,598	59.29	9.53	4.01
A[C→T]G	2,240,435	53.82	13.68	5.38
A[C→T]T	1,170,191	60.86	9.51	3.88

**Table 2.** Mean Tajima’s D (left) and *D*_−2_ estimator (right) for subtypes in each mutation group against subtypes not in group.

	Tajima’s D—No CpGs			*D*_−2_ Estimator—No CpGs		
Mutation group	Group mean	Non-group mean	*P*-value	Group mean	Non-group mean	*P*-value
Weak to strong (A→C, A→G)	−2.004	−2.058	2.2e−4	−0.243	−0.253	0.399
Indifferent (A→T, C→G)	−2.048	−2.033	0.387	−0.243	−0.252	0.450
Strong to weak (C→A, C→T)	−2.071	−2.024	2.2e−3	−0.264	−0.243	0.080

*P*-values computed from two-sample *t*-test. CpG subtypes were excluded from analysis.

Tajima’s D, a summary statistic of the entire high-dimensional genome-wide AFS, ranged from −2.19 to −1.50 across the 96 subtypes (Supplementary Fig. S1 in File S2). Uniformly negative values reflected the excess of rare variation observed. Even within a single mutation type there was variation in Tajima’s D when further considering adjacent nucleotides. For example, among C→G mutation types, Tajima’s D ranged from −2.19 to −1.50, with three CpG subtypes G[C→G]G (–1.50), A[C→G]G (−1.52), and T[C→G]G (−1.74) having clear lower outlier Tajima’s D values. Similar to comparisons in the singleton proportions, even outside CpG subtypes there existed variation in Tajima’s D (e.g. A→G mutation types ranged from −2.09 to −1.83). Substantial differences in both the proportions of singleton–tripleton proportions and Tajima’s D across the 96 3-mer subtypes summarizes heterogeneity in the AFS across sites.

### Comparison of the AFS across mutation subtypes to identify evolutionary forces driving AFS heterogeneity

**Fig. 3.**
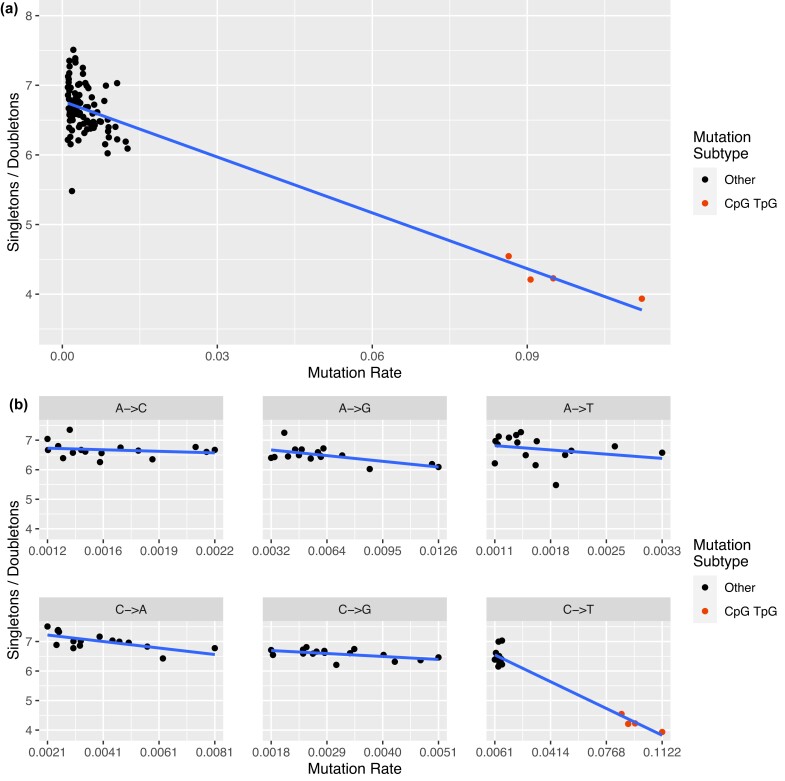
Correlation between the ratio of singletons to doubletons by the estimated mutation rate from extremely rare variants. a) Correlation of all 96 mutation subtypes. b) Correlation by six mutation types.

When assessing the relationship between mutation rates and the singleton to doubleton ratio, we found the ratio of singletons to doubletons was highly negatively correlated with the estimated singleton-derived mutation rates3 across the 96 mutation subtypes (*ρ* = −0.84, *p* = 2.2*e* − 16) (Fig. 3). As previously mentioned, sites with higher mutation rates are more susceptible to recurrent mutations. To assess whether the signal was driven by the CpG TpG sites with their order of magnitude higher mutation rates, we repeated the analysis after removing the 4 CpG TpG subtypes and found the ratio of singletons to doubletons was still negatively correlated with the estimated singleton-derived mutation rates (Carlson *et al.* 2018) across the 96 mutation subtypes (*ρ* = −0.35, *p* = 6.1*e* − 4). We further stratified subtypes by the six mutation types, observing consistently negative correlations between *ρ* = −0.21 and *ρ* = −0.97 (Supplementary Table S1 in File S2). Four of the six correlations (A→G, C→A, C→G, and C→T) were statistically significant even though each correlation is based on only 16 observations.

When comparing Tajima’s D and our *D*_−2_ statistic across subtypes to identify signals of gene conversion on the AFS, Tajima’s D ranged from [ − 2.19, − 1.84] (Supplementary Fig. S1 in File S2) while our *D*_−2_ statistic ranged from [−0.44, 0.10], with a strong correlation between the two statistics across subtypes (*ρ* = 0.76, *p* = 2.2*e* − 16). Under the null, we observed that *D*_−2_ was asymmetric with a heavier positive tail suggesting that utility of this statistic beyond summarizing the AFS shape without singletons and doubletons may be limited (Supplementary Fig. S2 in File S2). We grouped non-CpG mutation subtypes into weak to strong (WS), strong to weak (SW), and indifferent variants with each category having 32 subtypes and compared the mean Tajima’s D between groups (Table 2). We observed the smallest average Tajima’s D in SW (−2.071), followed by indifferent (−2.048) and WS (−2.004). Comparing each category against the mean of the other two categories, we found a statistically significant difference in means for WS vs SW and indifferent (*p* = 2.2*e* − 4, *t*-test) and SW vs WS and indifferent (*p* = 2.2*e* − 3, *t*-test). The “more positive” mean Tajima’s D for WS subtypes compared to non-WS indicated an excess of intermediate frequency variants, which is consistent with a model of gBGC where low-frequency S alleles get transmitted more often than expected and thus reach intermediate allele frequency more frequently. Similarly consistent with expectations under gBGC, the mean Tajima’s D was “more negative” for SW subtypes compared to non-SW.

As Tajima’s D is strongly dependent on the number of singletons and doubletons, the effect of gBGC may be confounded by mutation rate heterogeneity distorting primarily the singleton and doubleton counts. To limit the effect of mutation rate heterogeneity, we repeated the analysis using our *D*_−2_ statistic that ignores all singletons and doubletons in its calculation. We again observed the smallest Tajima’s D in SW followed by indifferent and WS (though indifferent and WS had very similar values) (Table 2), which was consistent with the hypothesized effect of gBGC on the AFS. When comparing each category against the mean of the other two categories, we observed a weaker signal of gBGC as only the SW comparison had a borderline statistically significant difference in mean Tajima’s D (*p* = 0.080, *t*-test) compared with WS and indifferent.

### Effect of heterogeneity in the genome-wide AFS across subtypes on demographic inference

**Fig. 4.**
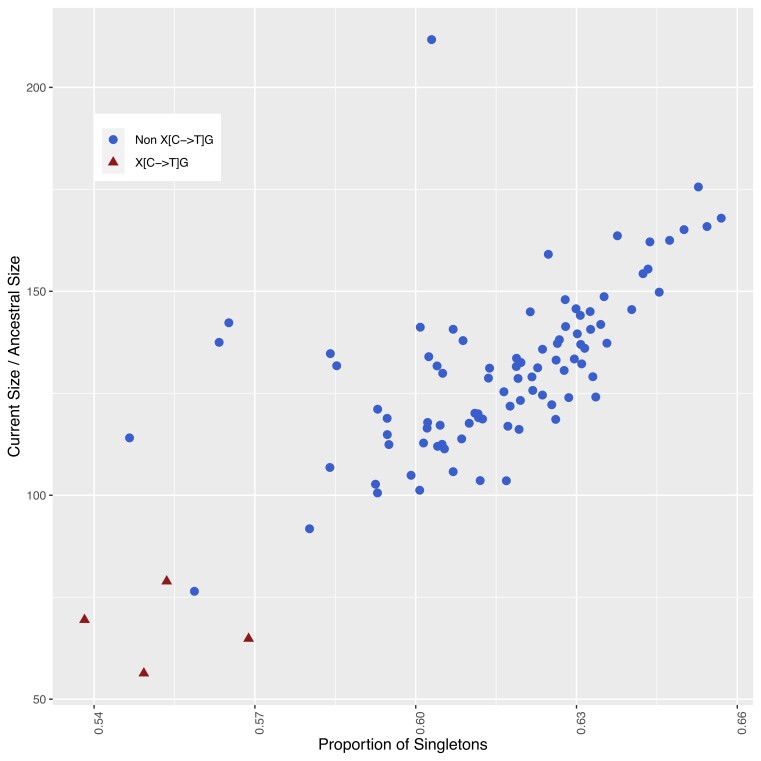
Scatterplot of inferred relative growth by the proportion of singletons for each of the 96 mutation subtypes’ AFS. CpG TpG sites denoted by triangle points have outlier higher mutation rates driving lower proportion of singletons.

Inferred demographic parameters varied drastically when running *δaδi* separately across the 96 distinct mutation subtype-specific genome-wide AFS. For the exponential growth model (Fig. 1), estimates of the ancestral effective population size derived from the inferred population genetics parameter *θ* varied 2-fold from 5062.99 to 10518.87 across the 96 subtype’s genome-wide AFS. Similarly, estimates of the time at which the ancestral constant population size started growing varied from 96.53 to 206.02 generations across subtypes (see Supplementary File S1). There was a strong correlation (*ρ* = 0.70, *p* = 2.06*e* − 15) between the genome-wide proportion of singletons for a given subtype and its inferred relative growth (Fig. 4). This is expected since the singleton count should be highly informative of growth rate and time since growth under an exponential growth model of human populations (Gao and Keinan 2014; Keinan and Clark 2012). CpG TpG sites have an overall lower proportion of singletons due to their higher mutation rate causing recurrent mutations, and thus had smaller inferred relative growth. However, two non-CpG subtype A[A→G]G and T[A→T]A had a similarly low proportion of singletons (<57%) and smaller inferred relative growth (114.07 and 76.44). In addition, the subtype G[A→G]G had an outlying higher inferred relative growth (211.71) given its proportion of singletons (0.60%) as compared to the trend of other subtypes.

For the constrained three-epoch model, inferred parameters across subtypes similarly varied drastically with the total time since the ancestral population first changed ranging from 7,530 to 824,292 generations, relative bottleneck depth ranging from 0.07 to 0.99, and relative recovery ranging from 3.77 to 90.43. Conclusions about the existence of a historical bottleneck varied across subtype inference, with four subtypes suggesting a nonexistent or very small bottleneck (less than a 15% decrease in effective population size). The remaining subtypes suggested a moderate to severe bottleneck with population contractions widely ranging from 40% to 93%. Excessively large times and recovery post bottleneck were likely driven by model constraints which forced a bottleneck to occur.

### Effect of heterogeneity in the local composition of mutation subtypes on the regional AFS

**Fig. 5.**
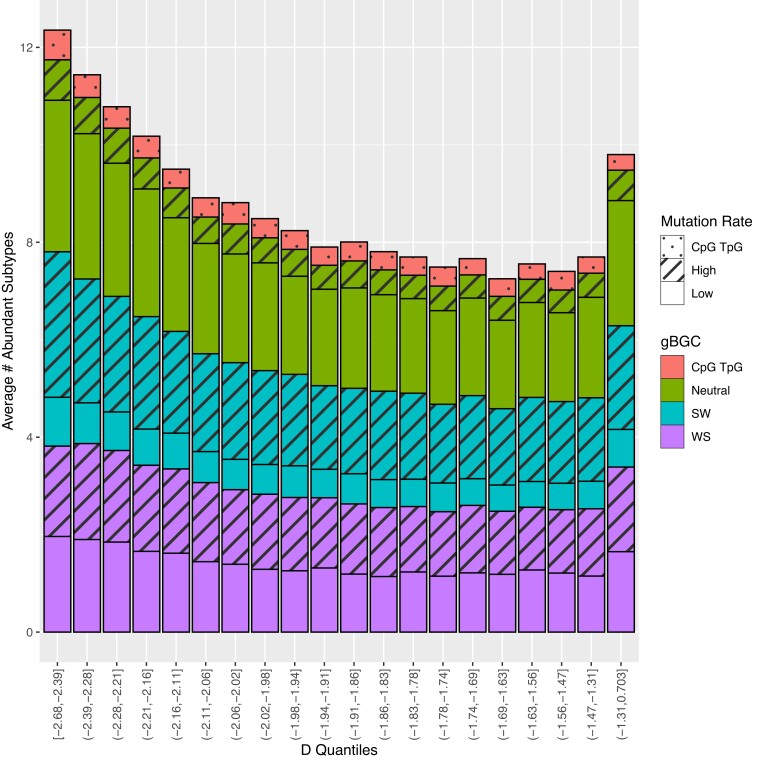
Average number of abundant subtypes in windows stratified by 5% Tajima’s D quantiles. Number of abundant subtypes further broken down into direction of gene conversion (strong to weak vs weak to strong) and mutation rate (low vs high).

In our regional analysis to assess whether local heterogeneity in subtype composition in a 100 kb windows shaped the regional AFS, we observed a general non-independence in the distribution of variants and Tajima’s D. Windows were first characterized as “abundant” in a given subtype for each of the 96 subtypes subtype if their proportion comprising the local AFS fell in the top 10% of windows genome wide (Fig. 2). After separating windows into 5% quantiles based off Tajima’s D, windows in the lowest and highest 5% of Tajima’s D quantile had on average more subtypes out of 96 with extreme abundances (12.35 and 9.79, respectively, vs 7.95) compared to the median D quantile average (Fig. 5), suggesting a more extreme composition of mutation subtypes in windows falling in the tails of the genome-wide Tajima’s D distribution. When stratifying abundant subtypes by direction of gene conversion and low/high mutation rate, we found no observable trends in proportions of each category across D quantiles (Supplementary Fig. S3 in File S2).

**Fig. 6.**
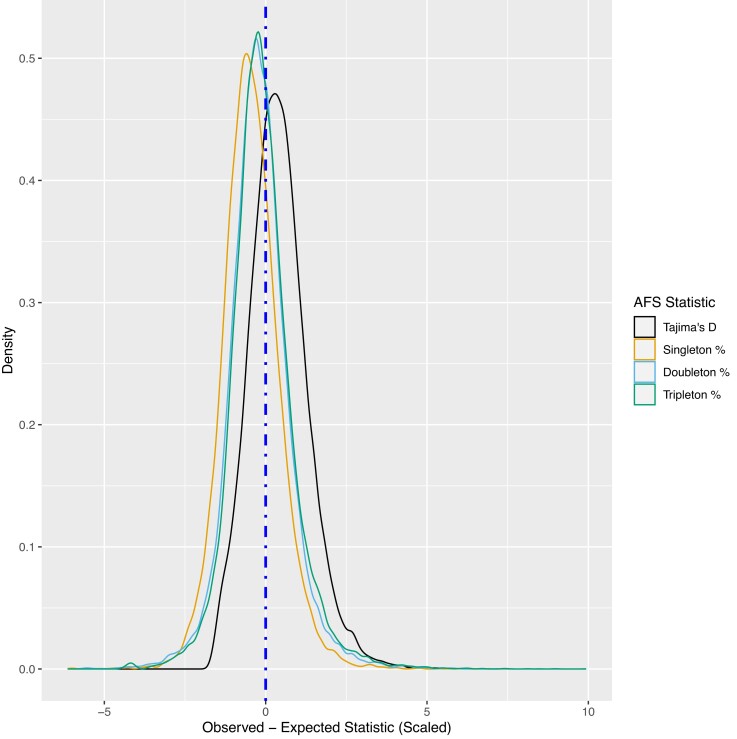
Difference in observed AFS statistics vs expected (MST genome-wide statistic weighted by counts of sites in local window), standardized for comparison across statistics. Dotted vertical line denotes zero, the difference if the local subtype composition perfectly determined the observed statistic.

We compared four observed local AFS statistics (Tajima’s D, % singletons, % doubletons, and % tripletons) to their expected value (see methods) to quantify the non-independence of local subtype composition and Tajima’s D in shaping the regional AFS. The mean (standard deviation) for observed AFS statistic across windows for Tajima’s D, % singletons, % doubletons, and % tripletons was −1.883 (0.335), 0.584 (0.037), 0.096 (0.015), and 0.039 (0.009). Similarly, expected local AFS statistics had means (standard deviation) of −2.036 (0.004), 0.603 (0.004), 0.099 (0.002), and 0.040 (0.001), with small standard deviations suggesting low variability in local subtype composition across most 100Kb windows. When compared, the mean (standard deviation) of the difference between observed AFS statistic and expected AFS Statistic across windows for Tajima’s D, % singletons, % doubletons, and % tripletons was 0.153 (0.335), −0.019 (0.038), −0.003 (0.015), and −0.001 (0.009) respectively (Fig. 6). Comparable standard deviations in the differences to the observed values alone were consistent with small variability in the expected values, with mean differences half a standard deviation or less from zero suggesting a role of local subtype composition shaping the regional AFS. Fewer observed variants at the rarest frequencies than expected may be driven by local genomic factors such as late replicating regions that elevate mutation rates outside subtype composition (Stamatoyannopoulos *et al.* 2009; Gaboriaud and Wu 2019).

From the GEE model directly regressing, the expected local AFS statistic and local genomic factors on the observed local AFS statistic, we found each expected local AFS statistic was a significant predictor despite low variability in their values. A 0.10 increase in expected Tajima’s D corresponded to a 0.285 (*p* = 4.47*e* − 03) increase in the observed Tajima’s D. Similarly, a 0.10 increase in expected % singletons, % doubletons, and % tripletons corresponded to a −0.085 (*p* = 1.85*e* − 05), 0.105 (*p* = 1.54*e* − 15), and 0.100 (*p* = 4.96*e* − 04) change in the corresponding observed AFS statistic on average (Supplementary Table S2 in File S2). A negative coefficient for the expected % singletons was inconsistent (Supplementary Fig. S4 in File S2), though could be explained by low variability in the expected values or negative correlations between CpG substitution rate and GC content. Furthermore, recombination rate was highly associated with the observed Tajima’s D (=0.045, *p* = 5.48*e* − 49), % singletons (=−0.006, *p* = 1.24*e* − 57), and % doubletons (=0.000, *p* = 1.92*e* − 08), while GC percent was only associated in the observed Tajima’s D model (=−0.394, *p* = 2.81*e* − 04). Overall, significant associations for all expected local AFS statistics, recombination rate, and GC content on the observed local AFS statistic values suggest that local mutation subtype composition shapes the regional AFS in conjunction with local genomic factors.

## Discussion

Our work uses large sample whole-genome sequencing to assess how the AFS differs across variant subtypes and identify biological factors driving AFS heterogeneity. While previous work has studied AFS heterogeneity mainly among the six single base mutation types (A→C, A→G, etc.), our results indicate increased heterogeneity when considering immediate flanking markers (especially for the rarest frequency singleton and doubleton variants). We further extend the effect of biological factors (hypermutability of certain motifs and biased gene conversion) on the AFS from 1-mer variants to 3-mers considering adjacent nucleotides. In particular, 3-mer motifs with higher mutation rates exhibited a lower ratio of singletons to doubletons while motifs, depending on direction of gene conversion, had an increase in either low or intermediate frequency variants as quantified by Tajima’s D and our proposed *D*_−2_ statistic removing singletons and doubletons. While conclusions of signals for gene conversion were consistent with our *D*_−2_ statistic, we note attenuated differences across groups are likely explained by a loss of power as the singleton and doubleton counts are very informative in D-type statistics.

We first demonstrated the effect of AFS heterogeneity across subtypes on demographic inference through considering the case of a single subtype comprising the entire genome-wide AFS. Under this scenario, inferred parameters differed drastically across subtypes and resulted in differing conclusions. Under the three-epoch population model, certain subtype specific AFS inferred a strong bottleneck or no bottleneck at all. Similarly, for an exponential growth model, growth rates varied drastically with a strong correlation between the singleton proportion and relative growth. CpG TpG sites inferred a lower relative growth due to having proportionally fewer singletons (driven by their higher mutation rates causing recurrent mutations). However, the subtypes A[A→G]G and T[A→T]A had a similarly smaller singleton proportion and also inferred lower growth. While CpG TpG sites are sometimes excluded from analysis as expected outliers (Excoffier *et al.* 2013), these are subtypes that would normally not be considered for exclusion.

Similarly, we found that regional AFS were also affected by AFS heterogeneity across mutation subtypes. Regions in the tails of the empirical distribution of Tajima’s D tend to have more extreme composition of mutation subtypes than windows near the median, though we saw no evidence that this local composition of mutation subtypes across Tajima’s D quantiles is driven by specific biological processes influencing the genome-wide AFS (gBGC and mutation rate heterogeneity). Mean differences between expected and observed local AFS statistics within half a standard deviation or less of zero suggest local heterogeneity in subtype composition plays a role in shaping the regional AFS. Larger mean differences (relative to standard deviations) for Tajima’s D and singleton proportion suggest the local subtype composition plays a relatively smaller role in shaping these statistics as compared to the doubleton and tripleton proportion. Furthermore, significant associations for expected statistics, recombination rate, and GC content in our GEE model suggest a combination of local subtype composition and genomic factors jointly shape the regional AFS.

Several potential limitations need to be considered when interpreting these results. First, AFS heterogeneity is driven by multiple factors acting concurrently, causing the identification of mutation rate heterogeneity and biased gene conversion to potentially confound one another. While we aimed to mitigate this issue using our newly derived *D*_−2_ statistic that removed singletons and doubletons when assessing signals of gene conversion, the effect of recurrent mutations driven by higher mutation rates detectably extends to higher allele frequencies. Second, correlations between estimated relative mutation rates and statistics/estimates across subtypes may be confounded by the fact that mutation rates used were estimated from singletons even though estimated relative mutation rates are similar to rates estimated elsewhere. Lastly, our dataset used had a relatively lower average coverage (9.6x) given today’s deep standards (>30x). While many extremely rare variants likely went undetected during variant calling, our stringent quality control procedure (see Carlson *et al.* 2018) ensured analyzed variants across the distribution of allele frequencies were of high quality.

Despite potential confounding and sample limitations, our results thus demonstrate the challenges introduced by treating polymorphic sites as exchangeable in population genetic inference. From our genome-wide analysis, we can clearly see how model parameter estimates for demographic inference are sensitive to the subtype-specific AFS and suggest removing certain subtypes prior to analysis. While CpG TpG sites are sometimes already excluded due to their outlying nature (Excoffier *et al.* 2013), demographic models dependent on the singleton count should further consider excluding other subtypes with lower singleton proportions. A sensitivity analysis with and without subtypes removed can then reveal how much inference is being driven by said subtypes. Pouyet *et al.* (2018) came to a similar conclusion, finding sites under gBGC and background selection could infer different demographic parameters. For example, in a Yoruban population they found these sites underestimated the age of a bottleneck and overestimated the magnitude of expansion, but no such bias appeared in another population. This led the authors to conclude future studies should use a set of markers avoiding these evolutionary forces, though specific biases on inference may be hard to predict. From our regional analysis, we can see that a combination of the local composition of subtypes and genomic factors play a role in shaping the regional AFS. Thus, we recommend approaches to identify regions under selection by identifying outlier local AFS statistics (i.e. Tajima’s D) to perform a post hoc analysis and consider whether a local region with an outlier Tajima’s D has a distribution of sites in the region comparable to the rest of the genome. Furthermore, we recommend considering whether the outlier D region is in a late replicating region [known to alter both the mutation rate and subtype composition (Kenigsberg *et al.* 2016)] or has an outlier recombination rate(as been suggested by Pouyet *et al.* (2018). If the region has a unique distribution of sites or is subject to one of the above specified local genomic factors, care may be needed in interpreting results of the analysis. Future work could potentially investigate adjusting either the overall AFS or the inference method itself to account for the composition of mutation subtypes in the sample prior to analysis.

Our findings about allele frequency heterogeneity imply that even non-AFS inference frameworks could bias inference by failing to differentiate between sites. For example, the coalescent-based methods PSMC (Li and Durbin 2011) and the Singleton Density Score (Field *et al.* 2016) treat sites as interchangeable. PSMC assumes a constant mutation rate in a window while the Singleton Density Score is reliant on distances to the nearest singleton, and thus both methods may be vulnerable to local regions being abundant in sites with outlying mutation rates or singleton proportions. As a result, we believe population genetics methods across multiple frameworks could benefit by carefully considering the unique evolution of mutation subtypes over time (Barroso and Dutheil 2021).

One benefit of the present era of population genetics is the availability of very large samples with deep genotyping. However, the same large sample sizes also amplify subtle population genetic effects that can mislead attempts at inference. As large samples provide an abundance of variants available for population genetic inference, it is both feasible and advisable to assess the robustness of inference results to these effects and to adjust the inference accordingly.

## Supplementary Material

jkad035_Supplementary_Data

## Data Availability

All datasets, scripts, and output from the analysis are available here: https://zenodo.org/record/7443570#.Y5t2yS1h1pQ. See URL for a detailed description of directories and files in zipped tar file. Included are relevant software/package to install as well as instructions on reproducing analysis. A description is also available here: https://github.com/kliao12/AFS_subtype_analysis. In addition, an R script to compute our *D*_−2_ estimator from any input AFS can be found here. Supplementary material are available at G3 online.
