# Supplementary material for: The effect of mutation subtypes on the allele frequency spectrum and population genetics inference
Source: G3 (Bethesda). 2023 Feb 10;13(4):jkad035. doi: 10.1093/g3journal/jkad035 (PMC10085755; doi:10.1093/g3journal/jkad035)
Supplement: jkad035_Supplementary_Data [file jkad035_supplementary_data.zip › File_S2_G3-2022-404001.pdf]

Supplementary Material for Effect of Mutation Subtypes on the Allele Frequency Spectrum and Population Genetics Inference

Derivation of New D_{-2} Estimator

We sought to derive a Tajima's D type statistic that removed the contribution of singletons and doubletons. The traditional Tajima's D is formulated as the difference of two unbiased estimates of θ: Mean pairwise difference (θ_π) and Watterson's estimator (θ_W) based on the total number of segregating sites:

$$D = \frac{\theta_\pi - \theta_W}{\sqrt{var(\theta_\pi - \theta_W)}}$$
$$\theta_\pi = \binom{n}{2}^{-1}\Big[\sum_{i=1}^{n-1}\eta_i i(n-i)\Big]$$
$$\theta_W = \frac{S}{h_n}$$

Here S is the number of segregating sites, n is the haploid sample size, and $h_n = \sum_{i=1}^{n-1}\frac{1}{i}$. For our novel D-2 statistic, the numerator takes the form of the traditional D estimator with the singleton and doubleton contributions subtracted from Mean Pairwise Difference and Watterson's Estimator. We then reweight the resulting estimators to both be unbiased estimators of θ.

1) MPD without singletons and doubletons. Note: we know from coalescent theory that under a neutral population $E[\eta_1] = \theta$ and $E[\eta_2] = \frac{\theta}{2}$.

$$\text{Let } \pi^* = \binom{n}{2}^{-1}\Big\{\Big[\sum_{i=1}^{n-1}\eta_i i(n-i)\Big] - \eta_1(n-1) - 2\eta_2(n-2)\Big\}$$
$$\begin{aligned}
E[\pi^*] &= E\Big[\binom{n}{2}^{-1}\Big\{\Big[\sum_{i=1}^{n-1}\eta_i i(n-i)\Big] - \eta_1(n-1) - 2\eta_2(n-2)\Big\}\Big] \\
&= E\Big[\pi - \binom{n}{2}^{-1}\eta_1(n-1) - \binom{n}{2}^{-1}\eta_2 2(n-2)\Big] \\
&= E[\pi] - \frac{n-1}{\binom{n}{2}}E[\eta_1] - \frac{2(n-2)}{\binom{n}{2}}E[\eta_2] \\
&= \theta - \frac{n-1}{\binom{n}{2}}\theta - \frac{2\theta(n-2)}{2\binom{n}{2}} \\
&= \theta\Big[1 - \frac{n-1}{\binom{n}{2}} - \frac{n-2}{\binom{n}{2}}\Big] \\
&= \theta\Big[1 - \frac{2}{n} - \frac{2(n-2)}{n(n-1)}\Big] \\
&= \theta\Big[\frac{n(n-1)}{n(n-1)} - \frac{2(n-1}{n(n-1)} - \frac{2(n-2)}{n(n-1)}\Big] \\
&= \theta\Big[\frac{n^2-5n+6}{n(n-1)}\Big] \\
&= \theta\Big[\frac{(n-3)(n-2)}{n(n-1)}\Big]
\end{aligned}$$

Thus

$$\theta_{\pi-2} = \frac{n(n-1)}{(n-2)(n-3)}\binom{n}{2}^{-1}\Big\{\Big[\sum_{i=1}^{n-1}\eta_i i(n-i)\Big] - \eta_1(n-1) - 2\eta_2(n-2)\Big\}$$

Is an unbiased estimator of θ

2) Watterson's estimator without singletons η_1 and doubletons η_2. Note: we know from coalescent theory $E[S] = h_n\theta$.

$$\begin{aligned}
\text{Let } S^* &= S - \eta_1 - \eta_2 \\
&= E[S^*] = E[S - \eta_1 - \eta_2] \\
&= h_n\theta - \theta - \frac{\theta}{2} \\
&= \theta(a_n - \frac{3}{2})
\end{aligned}$$

Thus

$$\theta_{W-2} = \frac{S - \eta_1 - \eta_2}{h_n - \frac{3}{2}}$$

Is an unbiased estimator of θ

Our novel D_{-2} statistic then takes the form:

$$D_{-2} = \frac{\theta_{\pi-2} - \theta_{W-2}}{\sqrt{var(\theta_{\pi-2} - \theta_{W-2})}}$$

For the denominator, we use results from Fu and DurettFu (1995); Durrett (2008) to derive an analytical form. Let c_n^k be a weight vector of length n for estimator X_k. Test of neutrality statistics can be expressed in the general form as a weighted sum of the AFS:

$$X_k = \sum_i^{n-1} c_{n,i}^k \eta_i$$

Our modified Watterson's and MPD estimators retain the same functional form subtracting off singletons and doubletons. Thus, they can be expressed as a weighted sum where the first two weights are set to zero and the remaining are reweighted:

1) MPD expressed as weighted sum

$$\begin{aligned}
\theta_{\pi-2} &= \frac{n(n-1)}{(n-2)(n-3)}\binom{n}{2}^{-1}\Big\{\Big[\sum_{i=1}^{n-1}\eta_i i(n-i)\Big] - \eta_1(n-1) - 2\eta_2(n-2)\Big\} \\
&= \sum_i^{n-1} c_{n,i}^1 \eta_i
\end{aligned}$$

$$\text{where } c_{n,i}^1 = [0, 0, \frac{2i(n-i)}{(n-2)(n-3)}, ...]$$

2) Watterson's expressed as weighted sum

$$\begin{aligned}
\theta_{W-2} &= \frac{S - \eta_1 - \eta_2}{h_n - \frac{3}{2}} \\
&= \sum_i^{n-1} c_{n,i}^1 \eta_i
\end{aligned}$$

$$\text{where } c_{n,i}^2 = [0, 0, \frac{1}{h_n - \frac{3}{2}}, ...]$$

Durett Durrett (2008) in Chapter 2, equation 2.27 and 2.28 presents a closed form covariance for any two estimators expressed as weighted sums of the AFS (using Fu's Fu (1995) results of the AFS covariance) as:

$$
\begin{aligned}
cov(X_1, X_2) &= a_n\theta + b_n\theta^2 \\
a_n &= \sum_{i=1}^{n-1} \frac{c^1_{n,i}c^2_{n,i}}{i} \\
b_n &= \sum_{i,j} c^1_{n,i}\sigma_{ij}c^2_{n,j} \\
\sigma_{ij} \text{ with i=j} &= \begin{cases} B_n(i+1) & i < \frac{n}{2} \\ 2\frac{h_n-h_i}{n-i} - \frac{1}{i^2} & i = \frac{n}{2} \\ B_n(i) - \frac{1}{i^2} & i > \frac{n}{2} \end{cases} \\
\sigma_{ij} \text{ with i>j } &= \begin{cases} \frac{B_n(i+1)-B_n(i)}{2} & i+j < n \\ \frac{h_n-h_i}{n-i} + \frac{h_n-h_j}{n-j} - \frac{B_n(i)+B_n(j+1}{2} - \frac{1}{ij} & i+j = n \\ \frac{b_n(j)-b_n(j+1)}{2} - \frac{1}{ij} & i+j > n \end{cases} \\
\text{where } B_n(i) &= \frac{2n}{(n-i+1)(n-i)}(h_{n+1} - h_i) - \frac{2}{n-i}
\end{aligned}
$$

Thus, we can derive the analytical form for the variance of our estimator

$$
\begin{aligned}
var(\theta_{\pi-2} - \theta_{W-2}) &= var(\theta_{\pi-2}) + var(\theta_{W-2}) - 2cov(\theta_{\pi-2}, \theta_{W-2}) \\
&= cov(\theta_{\pi-2}, \theta_{\pi-2}) + cov(\theta_{W-2}, \theta_{W-2}) - \\
&\quad 2cov(\theta_{\pi-2}, \theta_{W-2})
\end{aligned}
$$

Where each term can be expressed using Fu's closed form of the covariance described above and the corresponding weight vector c^k_n:

$$
\begin{aligned}
cov(\theta_{\pi-2}, \theta_{\pi-2}) &= a_n\theta + b_n\theta^2 \text{ with } c^1_n = c^2_n = [0, 0, \frac{2i(n-i)}{(n-2)(n-3)}, ...] \\
cov(\theta_{W-2}, \theta_{W-2}) &= a_n\theta + b_n\theta^2 \text{ with } c^1_n = c^2_n = [0, 0, \frac{1}{h_n - \frac{3}{2}}, ...] \\
cov(\theta_{\pi-2}, \theta_{W-2}) &= a_n\theta + b_n\theta^2 \text{ with } c^1_n = [0, 0, \frac{2i(n-i)}{(n-2)(n-3)}, ...] \\
c^2_n &= [0, 0, \frac{1}{h_n - \frac{3}{2}}, ...]
\end{aligned}
$$

Each of the above covariances requires knowledge of θ and θ^2 which are not known. Instead, we use estimates derived from Watterson's estimator, similar to the original Tajima's D statistic(Durrett 2008):

$$
\begin{aligned}
\hat{\theta} &= \frac{S}{h_n} \\
\hat{\theta^2} &= \frac{S^2 - S}{h_n - g_n} \quad \text{where} \quad g_n = \sum_{i=1}^{n-1} \frac{1}{i^2}
\end{aligned}
$$

To assess the null distribution and type 1 error, we used fastsimcoal2 to simulate 2,000 neutral frequency spectra for two subtypes: A[A->C]A and A[C->T]G with the same haploid sample size ($n = 7112$) observed in our data. For each run, we simulated a 1Mb sequence passing as parameters the subtype's absolute mutation rates and effective population size (23,785 and 23,066 respectively, derived using Watterson's estimate of θ and $\theta = 4N_e\mu L$ where L is the number of subtype motifs across the genome). Across simulations for both subtypes, the mean D_{-2} value was roughly zero (-0.103 and -0.048) (Figure S2 in File S2) with T1E rates of 0.031 and 0.0375 (roughly assuming a standard normal under the null). We note the null distribution is right skewed with a negative median, which mirrors the distribution of Tajima D from Tajima's originating work Tajima (1989). Similarities in the distribution are expected because D_{-2} fundamentally weights the remaining AFS the same as Tajima's D, up to a constant. Furthermore, in large samples, the skewness of Tajima's D may exacerbate as WakeleyWakeley (2008) detailed the coefficient of variation, a standardized measure of dispersion, for the number of segregating sites approaches zero as n tends to infinity while the mean pairwise differences tends to $\sqrt{\frac{1}{3\theta} + \frac{2}{9}}$. Thus, the right skew in the distribution of D_{-2} was not surprising given our large simulated sample size.

Negative Relationship Between Expected and Observed Singleton Proportions Across 100Kb Windows

In our 100Kb window analysis to determine whether the local subtype composition plays a role in shaping the regional AFS, we surprisingly observed a negative coefficient in our GEE regression model for the singleton proportion (Table S2 in File S2). Plotting the expected singleton proportion vs observed singleton proportion confirms a negative trend (Figure S4 in File S2). This may be caused by low variability in the expected singleton proportions [0.58, 0.61], which may obscure an actual positive relationship between values. Another possible explanation is GC content is known to be negatively correlated with CpG substitution rateMugal and Ellegren (2011); Fryxell and Moon (2005). Regions of high GC content would have lower mutation rates than expected (based on local mutation subtype composition) and could counteract the effect of recurrent mutation and thus increase the singleton proportions. While GC content was included in our model, we may not have sufficiently captured the complexity between substitution rates, methylation, and GC content.

Literature cited

Durrett R. 2008. *Probability models for DNA sequence evolution. Probability and its applications*. Springer. New York, NY.

Fryxell KJ, Moon WJ. 2005. CpG mutation rates in the human genome are highly dependent on local GC content. Mol. Biol. Evol.. 22:650–658.

Fu YX. 1995. Statistical properties of segregating sites. Theor. Popul. Biol.. 48:172–197.

Mugal CF, Ellegren H. 2011. Substitution rate variation at human CpG sites correlates with non-CpG divergence, methylation level and GC content. Genome Biol.. 12:R58.

Tajima F. 1989. Statistical method for testing the neutral mutation hypothesis by DNA polymorphism. Genetics. 123:585–595.

Wakeley J. 2008. Coalescent theory: an introduction. roberts and company. Greenwood VillageWayne AF, Maxwell MA, Ward CG, Vellios CV, Wilson I, Wayne JC, Williams MR (2015) Sudden and rapid decline of the abundant marsupial Bettongia penicillata in Australia. Oryx. 49:175185Webb.

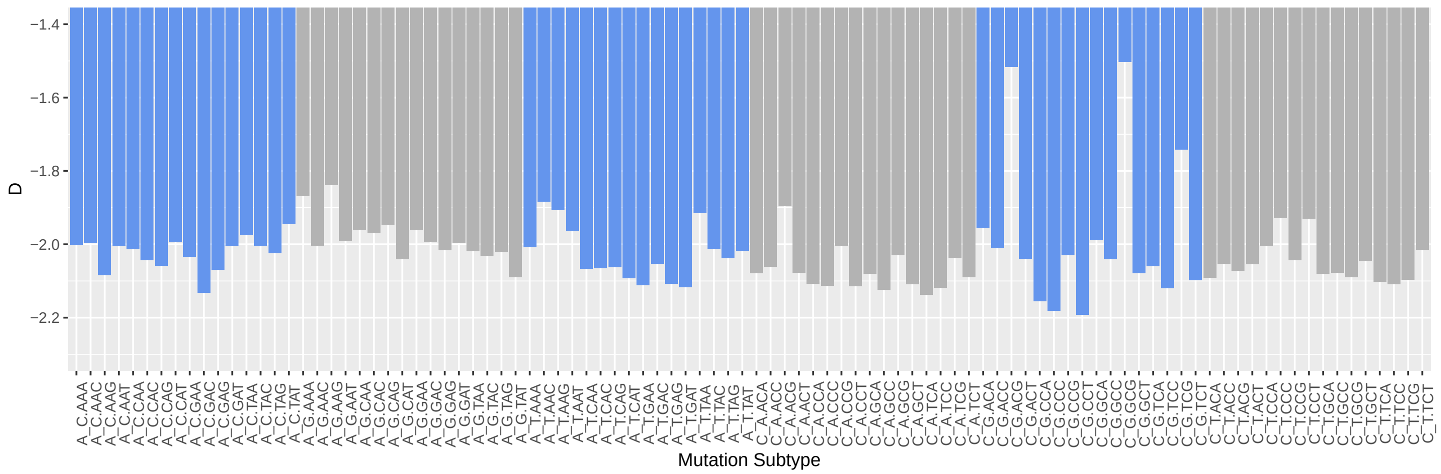

Figure S1 Bar plot showing Tajima's D computed for each of the 96 mutation subtypes' genome-wide allele frequency spectrum. Negative values across subtypes are consistent with recent explosive human population growth.

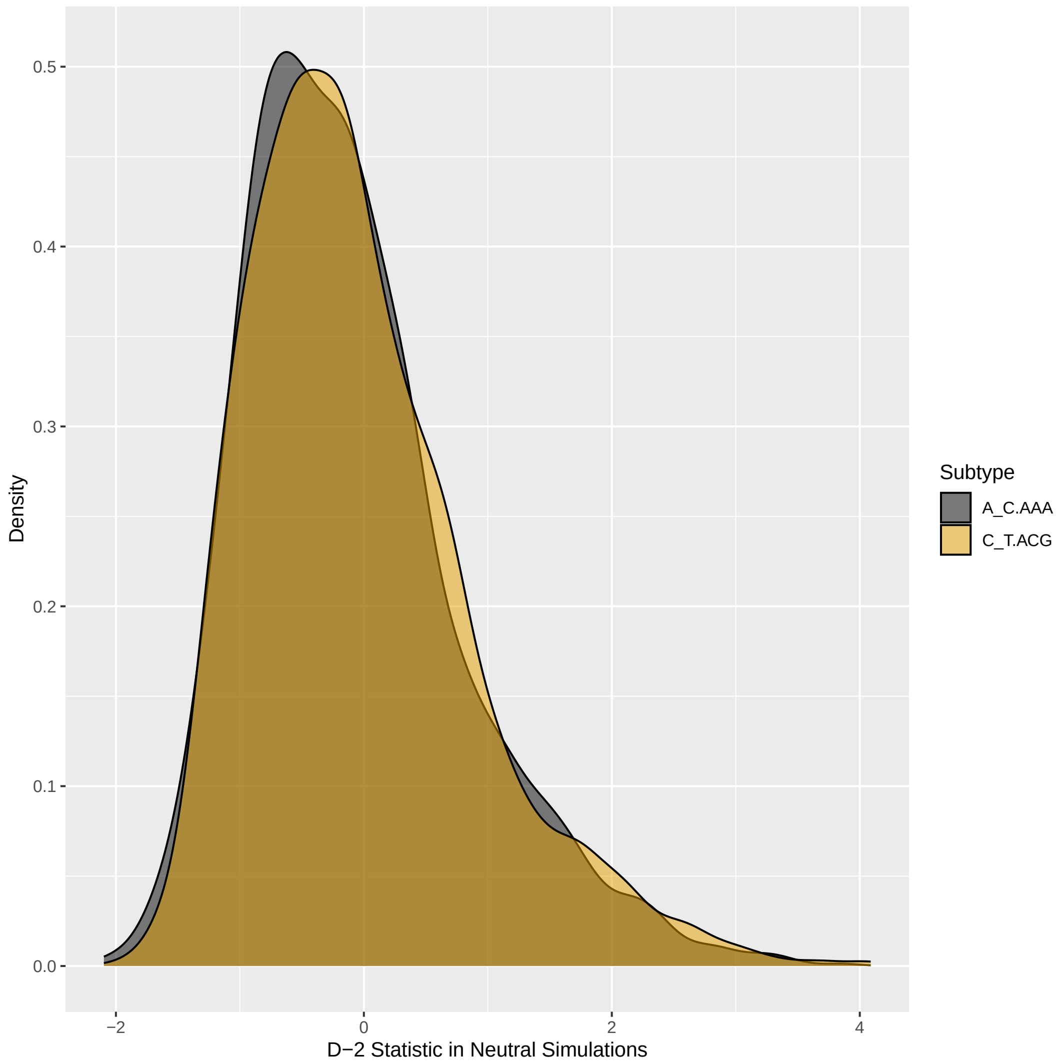

Figure S2 Null distribution for D-2 statistic across two subtypes: A[A->C]A and A[C->T]G. For each subtype, we simulated 2,000 neutral AFS using Fastsimcoal2 (see File S2).

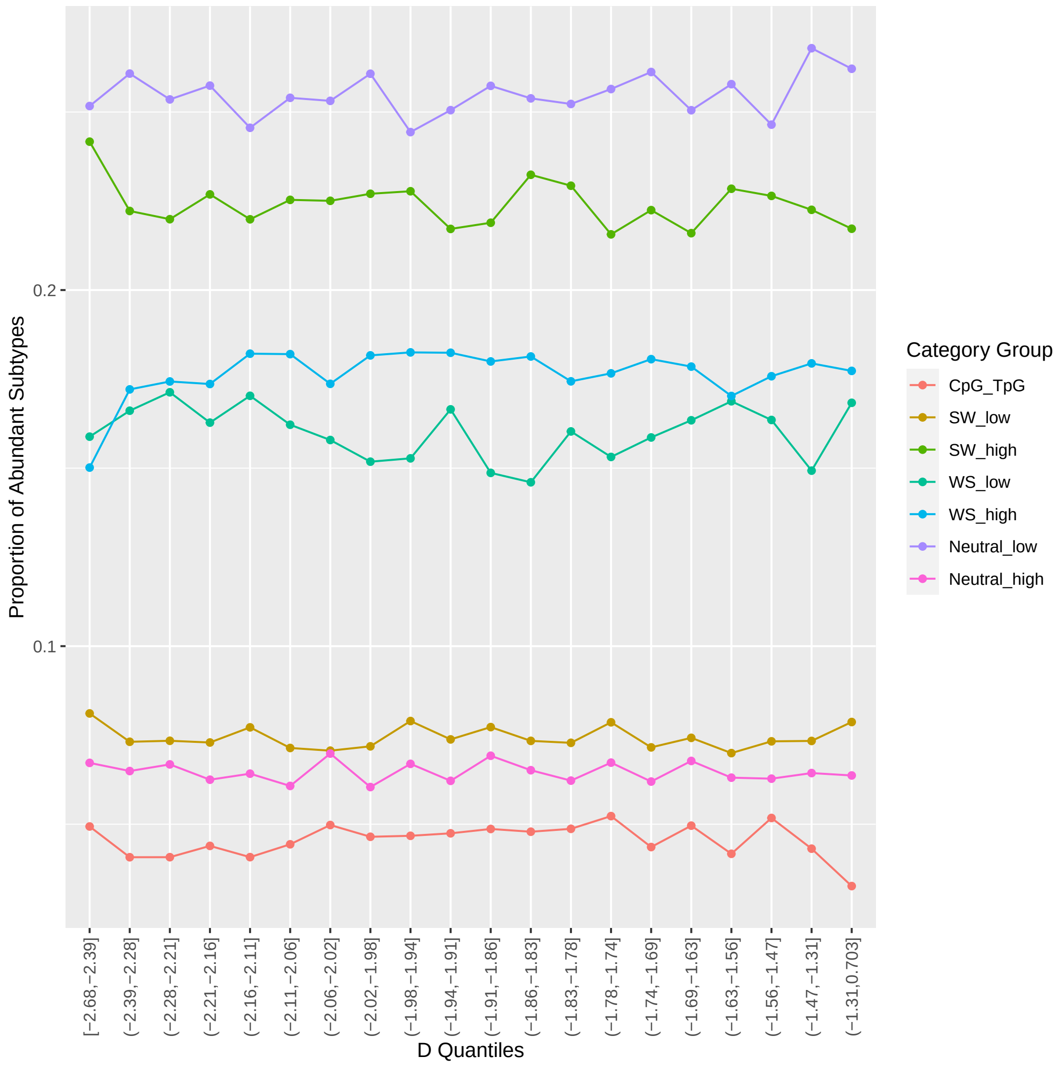

Figure S3 Line graph showing proportion of abundant subtypes in each Tajima's D quantile broken down by biased gene conversion x mutation rate heterogeneity category.

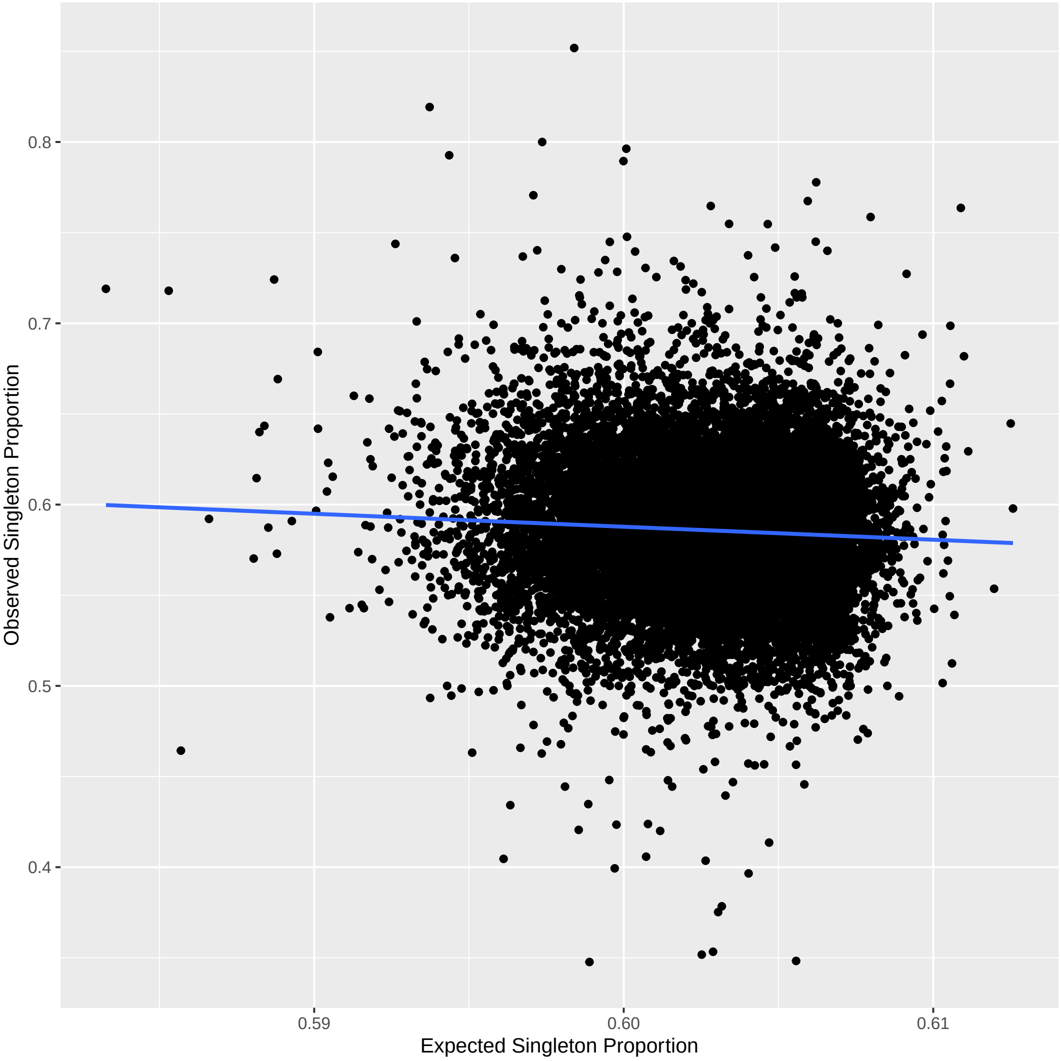

Figure S4 Scatter plot of negative relationship between expected and observed singleton proportion across 100Kb windows showing negative relationship.

Table S1 Correlation and p-value between singleton-derived mutation rates and singleton to doubleton ratio. Each mutation type has 16 distinct 3-mer subtypes in which correlation was computed.

Mutation Type	Correlation	P-Value
A->C	-0.21	0.44
A->G	-0.61	0.01
A->T	-0.26	0.33
C->A	-0.66	0.01
C->G	-0.54	0.03
C->T	-0.97	4.2e-10

Table S2 Regression output (β estimates and p values) from GEE analysis modeling observed local AFS statistics with expected statistics (defined as weighted mean of genome wide values, using counts of subtypes as weights) and adjusting for recombination rate and GC percent. Model run separately for each AFS statistic. P values used robust standard error estimates due to correlation in dependent variables across windows.

Covariates	Observed Statistic Dependent Variable			
	Model 1: Observed D	Model 2: Observed Singles	Model 3: Observed Doubles	Model 4: Observed Triples
Expected Statistic Estimate (p value)	2.853 (4.469e-03)	-0.845 (1.852e-05)	1.045 (1.536e-15)	1.003 (4.96e-04)
Recombination Rate Estimate (pvalue)	0.045 (5.479e-49)	-0.006 (1.237e-57)	0.000 (1.921e-08)	0.000 (0.573)
GC Percent Estimate (p value)	-0.394 (2.812e-04)	0.019 (3.719e-01)	-0.008 (3.132e-02)	-0.004 (0.183)
